# FASTMAP: Open-Source Flexible Atlas Segmentation Tool for Multi-Area Processing of Biological Images

**DOI:** 10.1523/ENEURO.0325-21.2022

**Published:** 2022-03-16

**Authors:** Dylan J. Terstege, Daniela O. Oboh, Jonathan R. Epp

**Affiliations:** Department of Cell Biology and Anatomy, Hotchkiss Brain Institute, Cumming School of Medicine, University of Calgary, Calgary, Alberta T2N 4N1, Canada

**Keywords:** atlas registration, brain mapping, open source

## Abstract

To better understand complex systems, such as the brain, studying the interactions between multiple brain regions is imperative. Such experiments often require delineation of multiple brain regions on microscopic images based on preexisting brain atlases. Experiments examining the relationships of multiple regions across the brain have traditionally relied on manual plotting of regions. This process is very intensive and becomes untenable with a large number of regions of interest (ROIs). To reduce the amount of time required to process multi-region datasets, several tools for atlas registration have been developed; however, these tools are often inflexible to tissue type, only supportive of a limited number of atlases and orientation, require considerable computational expertise, or are only compatible with certain types of microscopy. To address the need for a simple yet extensible atlas registration tool, we have developed FASTMAP, a Flexible Atlas Segmentation Tool for Multi-Area Processing. We demonstrate its ability to register images efficiently and flexibly to custom mouse brain atlas plates, to detect differences in the regional numbers of labels of interest, and to conduct densitometry analyses. This open-source and user-friendly tool will facilitate the atlas registration of diverse tissue types, unconventional atlas organizations, and a variety of tissue preparations.

## Significance Statement

Wide-scale studies examining relationships between different components of biological systems are becoming increasingly prevalent to diverse scientific questions. This process often requires the registration of biological samples to anatomic atlases. While progress has been made in the development of tools for the registration of mouse and rat brains, these tools are often inflexible to tissue type, tissue preparation, imaging plane, and atlas organization. To answer questions outside of this limited scope, it is imperative that analyses are flexible. To address this need, we have developed an open-source tool to register images to custom atlas plates. This custom registration tool facilitates atlas registration of diverse tissue types, unconventional atlas organizations, and a variety of tissue preparations across many scientific questions.

## Introduction

An overarching goal of behavioral neuroscience is to determine the relationship between brain structure and function. It has become recognized that many brain regions contribute to multiple functional processes. Consequently, understanding the regulation of functions by the brain requires exploration of the interactions between multiple brain regions. Histologic analysis of these relationships often requires the registration of anatomic regions from biological images to an atlas. Brain atlases exist for many species and are critical guides for delineating the many different regions of the brain. While the atlases themselves are excellent resources their application to an individual brain is challenging. Individual differences, as well as age, strain, and sex differences, mean that an atlas approximates but does not exactly map to an individual brain. Changes in brain shape and volume attributed to different tissue processing techniques further complicate the alignment of tissue samples with an atlas.

Manual tracing of a small number of regions based on guidance from an atlas is easily achievable but is very time consuming with many regions of interest (ROIs). To address this, several tools for brain atlas registration have been developed (Renier et al., 2016; Fürth et al., 2018; Lee et al., 2018; Claudi et al., 2020). These tools reduce the time required to process multiple regions in a dataset but are often limited to a specific model species, age, or tissue type. Imaging orientation and modalities are also often inflexibly linked to the analysis tool. Furthermore, the hierarchical level of atlas organization in these tools is generally fixed and for the user to register a single region to their image, they must align all regions to their image. This general lack of flexibility limits or complicates the application of many registration tools.

Existing studies which have applied atlas registration have used highly variable numbers of regions (Wheeler et al., 2013; Tanimizu et al., 2017; Abs et al., 2018; Silva et al., 2019; Doucette et al., 2020; Scott et al., 2020; Sun et al., 2020) tissue types (Asadulina et al., 2012; Randlett et al., 2015; Carson et al., 2016; Vetere et al., 2017; Asp et al., 2019; Choi et al., 2020), and labels (Sampedro-Piquero et al., 2018; González-Pardo et al., 2019). Flexible atlas registration facilitates analyses by providing those who would normally use a manual approach with a framework for efficient processing. Flexible registration is also beneficial because it allows users to better tailor registration to unique experimental designs.

We have developed a Flexible Atlas Segmentation Tool for Multi-Area Processing (FASTMAP) for efficient registration of biological images to user-generated, customized atlases. Using functions native to the free image analysis program ImageJ (Schneider et al., 2012), FASTMAP facilitates the segmentation of labels, registration of ROIs, and analyses of label density across user-defined atlases. Here, we demonstrate the versatility FASTMAP and apply the tool to several biological image sets. We show that FASTMAP can be flexibly applied to different imaging planes, various markers and can register higher and lower atlas hierarchies.

## Materials and Methods

### Animals

Mice used in the following experiments were bred in established colonies at the University of Calgary. Male tgCRND8 mice were used to assess the ability of FASTMAP to detect age-related changes in amyloid plaque distribution. Male C57BL6J mice, at either 12 weeks of age or embryonic day (e)16, were used for all other experiments. All mice were housed under standard laboratory housing conditions with *ad libitum* access to water and standard laboratory diet. All animal procedures were in accordance with the guidelines established by the Canadian Council for Animal Care and were approved by the University of Calgary Health Sciences Animal Care Committee.

### c-Fos labeling

Brain-wide c-Fos expression was induced during the recall of a contextually conditioned memory. One mouse was trained in a contextual fear conditioning task in a sound-attenuated chamber (Ugo Basile) with a grated floor through which three shocks (0.5 mA, 2 s) were delivered. A retention test was conducted 24 h later, and the mouse was transcardially perfused 90 min after the test. The mouse was perfused with 0.1 m PBS followed by 4% formaldehyde. The brain was extracted and postfixed for 24 h in 4% formaldehyde before being cryoprotected in 30%sucrose. The brain was serially sectioned in a sagittal plane with a thickness of 40 μm on a cryostat (Leica Biosystems) and stored at −20°C in antifreeze solution. Every 12th tissue section was incubated in a primary antibody solution, for 48 h, consisting of 1:2000 rabbit anti-c-Fos (RPCA-cFOS, EnCor Biotechnology Inc.). followed by a 24-h incubation in 1:500 donkey anti-goat Alexa Fluor 647 (AB_2338072, Jackson ImmunoResearch). Sections were then incubated in a 1:1000 dilution of 4′,6′-diamidino-2-phenylindole (DAPI) for 15 min before being mounted to slides and coverslipped using PVA-DABCO mounting medium.

Tissue was imaged using an Olympus VS120-L100-W slide scanner with an ORCA-Flash 4.0 camera and 10× objective. Fluorophores were imaged with DAPI and Cy5 filter cubes. Analysis was conducted on a subset of three sections across this brain, as a proof of concept.

### Vascular labeling

A FITC-albumin gelatin perfusion protocol was used to label vasculature and was performed as previously described (Lugo-Hernandez et al., 2017). After perfusion labeling and fixation, serial sagittal sections with a thickness of 100 μm were cut on a cryostat and stored at −20°C in antifreeze solution. Every 12th section was incubated in 1:1000 propidium iodide in 0.1 m PBS for 15 min before being mounted to glass slides and coverslipped with PVA-DABCO mounting medium.

Tissue was imaged using an Olympus FLUOVIEW FV3000 confocal microscope with 10× objective and digital zoom of 1.50× was applied to the *XY* imaging plane and Z-spacing was 5.33 μm. Detailed photomicrographs of the dentate gyrus were acquired using a 20× objective and digital zoom of 4.89× with Z-spacing of 1.27 μm.

### Amyloid labeling

CRND8 mice were used to assess the ability of FASTMAP to detect age-related changes in the regional density of fluorescently labeled β-amyloid across the brain (Azhar Chishti et al., 2001). To label amyloid plaques, mice were administered 7.5 mg/kg methoxy-X04 in 10% DMSO and 0.1 m PBS (Klunk et al., 2002).

Mice [3 months old (*n *=* *3), 5 months old (*n *=* *3), 10 months old (*n *=* *3)] were transcardially perfused 24 h after methoxy-X04 injections, with 0.1 m PBS followed by 4% formaldehyde. Brains were postfixed for 24 h in 4% formaldehyde. Fixed brains were cryoprotected in 30% sucrose. Sagittal sections 40 μm thick were cut on a cryostat and stored at −20°C in antifreeze solution. Every 12th section throughout the brain was incubated in 1:1000 propidium iodide in 0.1 m PBS for 15 min. Stained sections were mounted to glass slides and coverslipped with PVA-DABCO mounting medium.

Slides were imaged using an Olympus BX63 automated fluorescence microscope with a Hamamatsu ORCA-Fusion camera, 10× objective. Methoxy-X04-labeled amyloid deposits were imaged using a GFP filter cube at an exposure time of 110 ms while propidium iodide labeled cells were imaged using an RFP filter cube at an exposure time of 80 ms. Image stacks with Z-spacing of 4 μm were collected and maximum intensity Z-projections were saved as separate TIF images for each channel.

### Parvalbumin labeling

Brains from C57BL6J mice were serially sectioned in the coronal plane with a thickness of 40 μm on a cryostat. Every 12th tissue section was incubated for 48 h in a primary antibody solution consisting of 1:1000 guinea pig anti-PV (195005, Synaptic Systems), followed by1:500 donkey anti-guinea pig Alexa Fluor 647 (AB_2340476, Jackson ImmunoResearch) secondary antibody for 24 h. Sections were then incubated in a 1:1000 dilution of DAPI for 15 min before being mounted and coverslipped using PVA-DABCO mounting medium.

Slides were imaged using an Olympus BX63 automated fluorescence microscope with a Hamamatsu ORCA-Fusion camera and 10× objective. Images were collected as 40-μm image stacks with Z-spacing of 4 μm. Maximum intensity Z-projections were saved as separate images for each channel. Six thalamic reticular nuclei from three coronal sections each spaced by 480 μm along the anterior-posterior axis were traced by an experienced rater using ImageJ.

### Segmentation of fluorescent labels

Methoxy-X04-labeled amyloid plaques and FITC-labeled vasculature were segmented within FASTMAP using threshold commands in ImageJ. This command can be modified depending on the segmentation needs of the label of interest and could be removed entirely if the input image were already binarized using an object segmentation tool, such as *Ilastik* (Berg et al., 2019), as was the case with the images of c-Fos-labeled tissue sections.

### Sagittal mouse brain atlas generation and registration

Using the 2011 Allen Mouse Brain Atlas as a reference (Lein et al., 2007), custom atlas plates were generated. For the lower-level atlas, which was used in the vasculature and amyloid experiments, a total of 12 regions were drawn from sagittal reference atlas plates (Table 1). The c-Fos mapping experiment used a higher-level atlas with 63 regions (Table 2). Atlas plates were traced using ImageJ and saved as ROI sets through the ROI Manager applet. Each of the saved ROI sets corresponded to a unique plate in the Allen Mouse Brain Atlas. To map target images to these atlas plates, target images were sequentially loaded and FASTMAP prompted the user to identify which atlas plate most closely resembled each image. ROI sets corresponding to each selection were loaded, and regions were resized using a linear transform to the fit the dimensions of the target images. Regions were then sequentially adjusted using manual nonrigid free-form deformation to align with the target images.

**Table 1:** Regions included in the described lower-level atlases

Abbreviation	Region	Abbreviation	Region
AMY	Amygdala	MB	Midbrain
CB	Cerebellum	MY	Medulla
HB	Hindbrain	OLF	Olfactory bulbs
HPF	Hippocampal formation	PAL	Pallidum
HY	Hypothalamus	STR	Striatum
ISO	Isocortex	TH	Thalamus

List of the 12 regions and their associated abbreviations which were included in the custom lower-level neuroanatomical atlases.

**Table 2:** Regions included in the described higher-level atlases

Abbreviation	Region	Abbreviation	Region
ACA	Anterior cingulate area	PAG	Periaqueductal gray
ACB	Nucleus accumbens	PALc	Caudal pallidum
AHN	Anterior hypothalamic nucleus	PALm	Medial pallidum
AON	Anterior olfactory nucleus	PALv	Ventral pallidum
ATN	Anterior dorsal thalamus	PCG	Pontine central gray
CA1	Field CA1	PG	Pontine gray
CA2	Field CA2	PH	Posterior hypothalamus
CA3	Field CA3	PHY	Perihypoglossal nucleus
DG	Dentate gyrus	PMd	Dorsal premammilary
DMH	Dorsomedial hypothalamus	PMv	Ventral premammilary
DMX	Dorsal motor nucleus of the vagus nerve	PRN	Pontine reticular nucleus
EPI	Epithalamus	PRT	Pretectal region
FN	Fastigial nucleus	PVH	Paraventricular hypothalamus
FRP	Frontal pole	RCH	Retrochiasmatic area
GRN	Gigantocellular reticular nucleus	RN	Red nucleus
IC	Inferior colliculus	RSP	Retrosplenial cortex
ILM	Intralaminar nucleus	RT	Reticular nucleus of the thalamus
IO	Inferior olivary complex	SCm	Motor superior colliculus
IRN	Intermediate reticular nucleus	SCs	Sensory superior colliculus
LDT	Laterodorsal tegmental nucleus	SF	Septofimbrial nucleus
LS	Lateral septum	SOC	Superior olivary complex
MARN	Magnocellular reticular nucleus	SPF	Subparafascicular nucleus
MDRN	Medullary reticular nucleus	SUB	Subiculum
MED	Medial dorsal thalamus	TRN	Tegmental reticular nucleus
MO	Somatomotor areas	TT	Taenia tecta
MOB	Main olfactory bulb	VENT	Ventral group of the dorsal thalamus
MPN	Medial preoptic nucleus	VERM	Vermal regions
MPO	Medial preoptic area	VI	Abducens nucleus
MRN	Midbrain reticular nucleus	VMH	Ventromedial hypothalamic nucleus
NTS	Nucleus of the solitary tract	VNC	Vestibular nuclei
ORB	Orbital area	VTA	Ventral tegmental area
OT	Olfactory tubercle		

List of the 63 regions and their associated abbreviations which were included in the custom higher-level neuroanatomical atlases.

### Generation of additional atlases

To demonstrate atlas flexibility, images were gathered of other tissue types and orientations. Images of sagittal, coronal, coronal tissue block, and horizontal sections were gathered of tissue from C57BL/6 mice. Mice were perfused, labeled with DAPI, and imaged with an Olympus BX63 automated fluorescence microscope as described above. Images of the coronal sections were also used to assess interrater reliability. Images of mouse embryonic tissue were collected from an e16 C57BL/6 mouse. Tissue was cleared using uDISCO as described previously (Li et al., 2018), and images were collected using a fluorescent light-sheet microscope equipped with an OLYMPUS MXV10 stereo microscope, an ANDOR Zyla 5.5 sCMOS camera, and lasers and drivers from LaVision BioTec (LaVision BioTec). Regions were delineated using the Allen Mouse Brain Atlas and Embryonic Mouse Brain Atlas as references.

### Assessment of registration reliability

The ability of FASTMAP users to reliably register ROIs was assessed by comparing registrations conducted by four independent raters who were naive to FASTMAP but who had prior familiarity with rodent neuroanatomy. C57BL/6 mice were perfused, labeled with DAPI, sectioned in a coronal plane and imaged with an Olympus BX63 automated fluorescence microscope as described above. Each independent rater registered a subset of five coronal DAPI channel images to a custom low-level mouse brain atlas. Area measurements for like regions were summed for each rater, with each region being present in two to five images. To assess the extent to which raters produced similar results, both the summed areas of like regions and the percent overlap for like regions was compared across raters. Percent overlap was calculated using ImageJ by combining corresponding traces from all raters and assessing the percent similarity between this combined trace and each individual trace. These same images were also registered using the commercial registration program *NeuroInfo* (Microbrightfield; Tappan et al., 2019).

### Technical requirements

FASTMAP is supported on macOS (tested 10.15.7) and Windows (tested Windows 10.0.19 042). It is operational on both ImageJ (version tested 1.52a; https://imagej.nih.gov/ij/download.html) and FIJI (version tested 1.53f; https://fiji.sc). Minimum system requirements for these programs are Windows XP and macOS 10.8 “Mountain Lion” with Java installed on each. Memory requirements are scaled relative to the size of the images; however, no issues were reported on any device with 8 GB of RAM or greater.

### Software accessibility

The software described in the paper is freely available online at https://github.com/dterstege/FASTMAP. The code is available as Extended Data 1. The source code is licensed under a GPLv3 license. The software can easily be adapted to use custom atlases developed by the end user. We have also included several premade atlases that may be used. Instructions and tutorials for use are available online.

10.1523/ENEURO.0325-21.2022.ed1 Extended Data 1 FASTMAP user guide. Full FASTMAP user guide posted to the GitHub repository, at https://github.com/dterstege/FASTMAP. Download Extended Data 1. Download Extended Data 1, ZIP file.

### Statistical analysis

Statistical analyses were conducted using Prism (version 9.1.0, GraphPad Software, LLC). To detect statistical significance, a *p*-value of 0.05 was set as the threshold for significance. To assess reliability, we calculated the Krippendorff’s α reliability coefficient, whereby values closest to 1 are most reliable (Shelley and Krippendorff, 1984).

### Data availability

Any data generated during the current study will be made available online at https://github.com/dterstege/PublicationRepo/tree/main/Terstege2022B.

## Results

### Atlas registration for the mapping of brain-wide c-Fos expression

**Figure 1.**
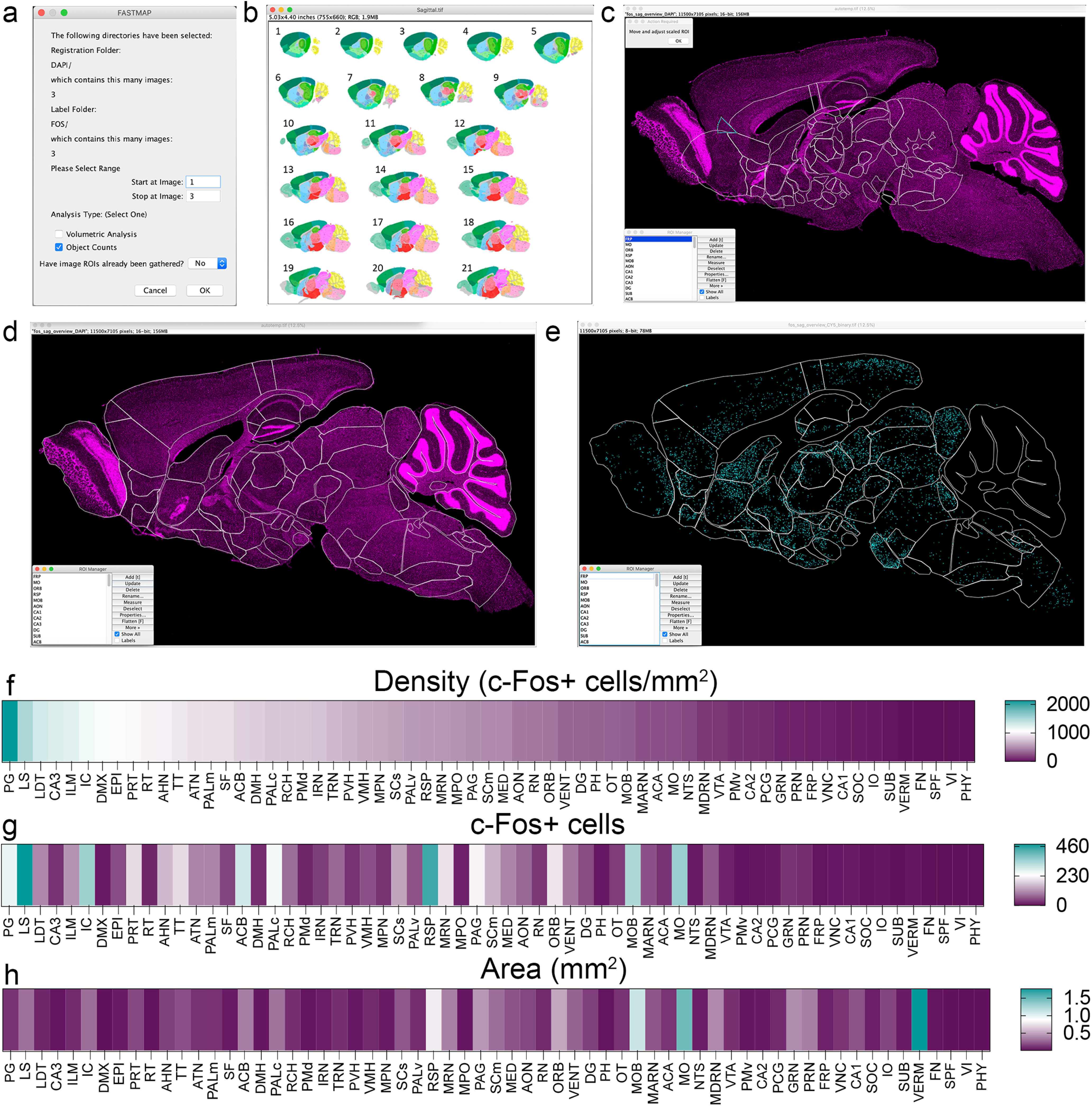
A tool for the flexible atlas registration of biological images and brain-wide mapping of a label of interest. When running the analysis pipeline, the user is presented with the opportunity to limit the range of images and to define whether to count labels of interest or apply a densitometry-based approach (***A***). The user will then be prompted to select which atlas plate most closely aligns with the image they are currently registering (***B***, thumbnail reference atlas images credited to the Allen Institute and the Allen Mouse Brain Atlas; Lein et al., 2007). The selected atlas plate then loads over the target image and individual regions resize sequentially to suit size of the target image (***C***). Regions are manually moved and adjusted to align with the target image (***D***). Once the alignment is correct, the registration is then applied to a binarized label of interest, in this case c-Fos-expressing cells (***E***). The regional density of c-Fos-expressing cells (***F***), the number of c-Fos-expressing cells (***G***), and the area of each region (***H***) can all be obtained as outputs using this analysis type. For program source code and user guides, see Extended Data 1. *Figure Contributions*: Dylan J. Terstege prepared the tissue, collected photomicrographs, and conducted the analysis.

To demonstrate the flexibility of FASTMAP, the tool was used to map brain-wide c-Fos expression during the recall of a contextually conditioned memory (Fig. 1). FASTMAP was directed to folders containing a subset of images of the c-Fos channel and corresponding DAPI channel images (*n *=* *3). The range of images to be registered within these directories was set and an analysis type was selected (Fig. 1*A*). The option “Volumetric Analysis” could be used to assess density by percentage of label in each region. For the c-Fos analysis, “Object Counts” was selected to count the number of c-Fos+ cells within each region. The atlas plate which most closely resembled each image was identified and loaded over its target image (Fig. 1*B*,*C*). Regions were resized automatically to suit the target dimensions before users were presented with the opportunity to manually adjust regions in a free-form manner to account for imperfections (Fig. 1*E*). The registration was then applied to the c-Fos channel, and regional c-Fos density, c-Fos counts, and areas were calculated (Fig. 1*E–H*).

### Reliability of registration based on DAPI labeling

**Figure 2.**
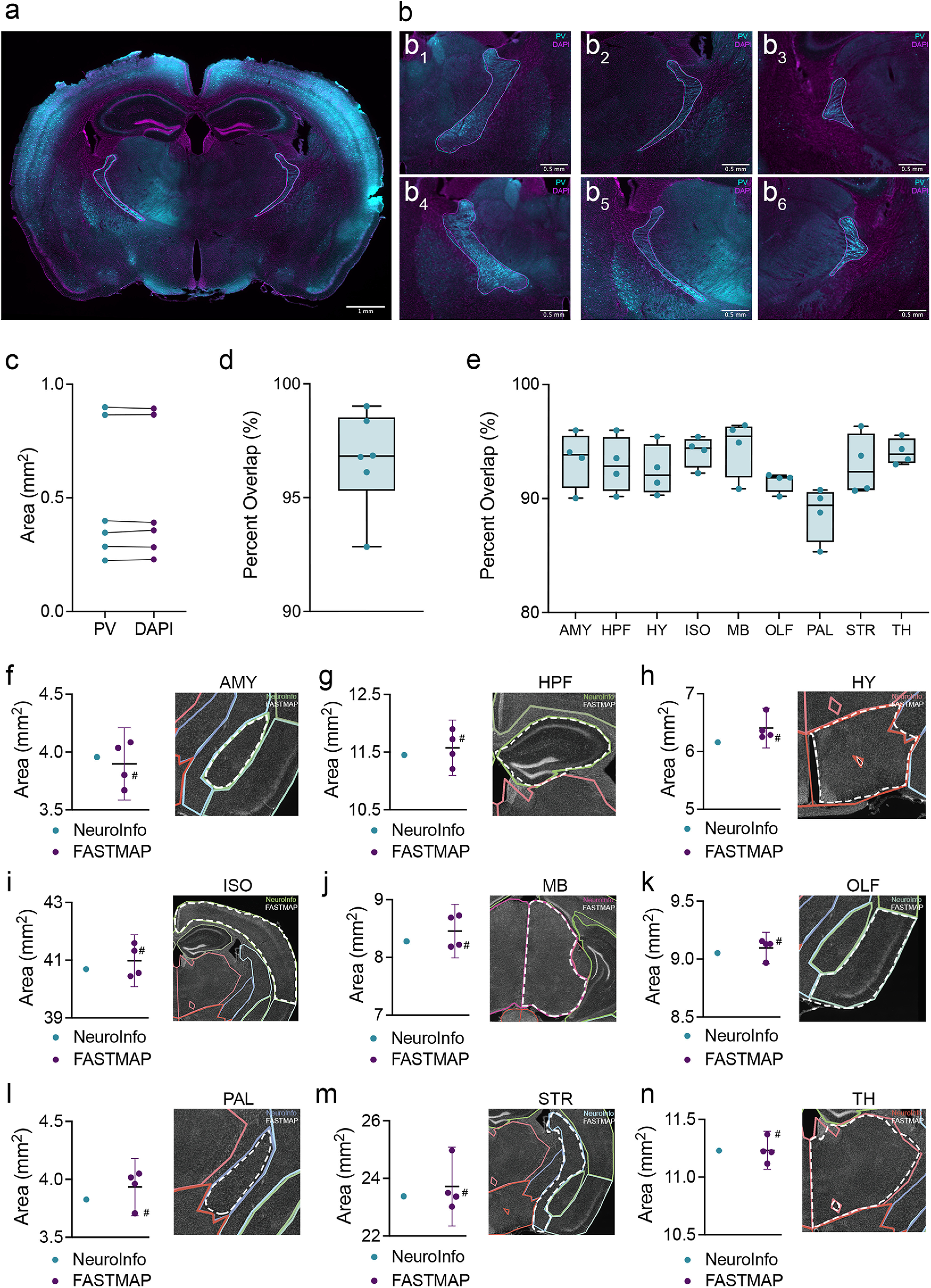
DAPI provides sufficient contrast for accurate and reliable neuroanatomical registration. ***A***, Representative photomicrograph of parvalbumin and DAPI staining from a coronal section of adult mouse brain. Overlay shows traced regions for each channel (parvalbumin = cyan, DAPI = magenta) ***B***, The thalamic reticular nucleus was traced across multiple sections based on parvalbumin (cyan) and DAPI (magenta) labeling. ***C***, There were no significant differences in area measurements of thalamic reticular nuclei traced from each imaging channel. ***D***, Areas traced using DAPI labeling as a reference overlapped with areas traced based on parvalbumin labeling by an average of 96.66%, with a range of 6.172% (92.84–99.012%). ***E***, Using a DAPI channel, independent raters (*n *=* *4) used FASTMAP to record the areas of the amygdalar areas (AMY), hippocampal formation (HPF), hypothalamus (HY), isocortex (ISO), midbrain (MB), olfactory cortex (OLF), pallidum (PAL), striatum (STR), and thalamus (TH) from a subset of images (*n *=* *2–5 per area) across an adult mouse brain. The extent to which the individual tracings overlapped with a summed composite trace was calculated to have median values of 93.83% for AMY, 92.85% for HPF, 92.07% for HY, 94.41% for ISO, 95.46% for MB, 91.82% for OLF, 89.41% for PAL, 92.34% for STR, and 93.89% for TH. Areas recorded from these tracings, summed across images for each region (***F–N***), did not differ from areas collected using the commercial registration tool *NeuroInfo*. Representative *NeuroInfo* registrations (colored outlines) and FASTMAP registrations (white outlines) are provided for each region. Representative FASTMAP registrations were selected for visualization based on which of the independent raters produced a summed area measurement (across all regions) closest to the median value among independent raters. The measurement that corresponds to the sample trace is indicated in each plot with an octothorpe (#). Data presented as individual matched datapoints (***C***), median ± max/min (***D***, ***E***), and mean ± 95%CI (***F–N***). *Figure Contributions*: Dylan J. Terstege prepared the tissue, collected photomicrographs, and conducted the analysis.

FASTMAP uses atlas-guided registration of an imaging channel such as DAPI staining. However, cell type-specific markers can also be useful in determining the boundaries of particular regions. For example, parvalbumin labeling may facilitate the delineation of the thalamic reticular nucleus (Fig. 2*A*,*B*). We used this approach to demonstrate the reliability of DAPI-based regional tracing in FASTMAP. In a series of images, the DAPI channel was first used to outline the thalamic reticular nucleus, then the same images were outlined using the parvalbumin labeled channel and areas were compared (Fig. 2*C*). While the area of the reticular thalamic nucleus expectedly varied along the anterior-posterior axis, there were no differences between sets of thalamic reticular nuclei areas registered using these channels (two-tailed paired *t* test, *t*_(5)_ = 0.025, *p *=* *0.98; Fig. 2*D*). The extent to which registered areas overlapped across tracing conditions was determined to be 96.7% on average (Fig. 2*D*).

Area measurements obtained using *NeuroInfo* were all within the 95%CI of independent FASTMAP users. These independent raters also displayed considerable interrater reliability using FASTMAP, with a high percentage of overlap between raters (Fig. 2*E*). Additionally, the median difference in area measurements between methods being only 1.59% (Fig. 2*F–N*). There was also considerable reliability in the measurements recorded by independent raters, indicated by a Krippendorff’s α of 0.99 for these areas.

### Regional distribution of vasculature in the adult mouse brain

**Figure 3.**
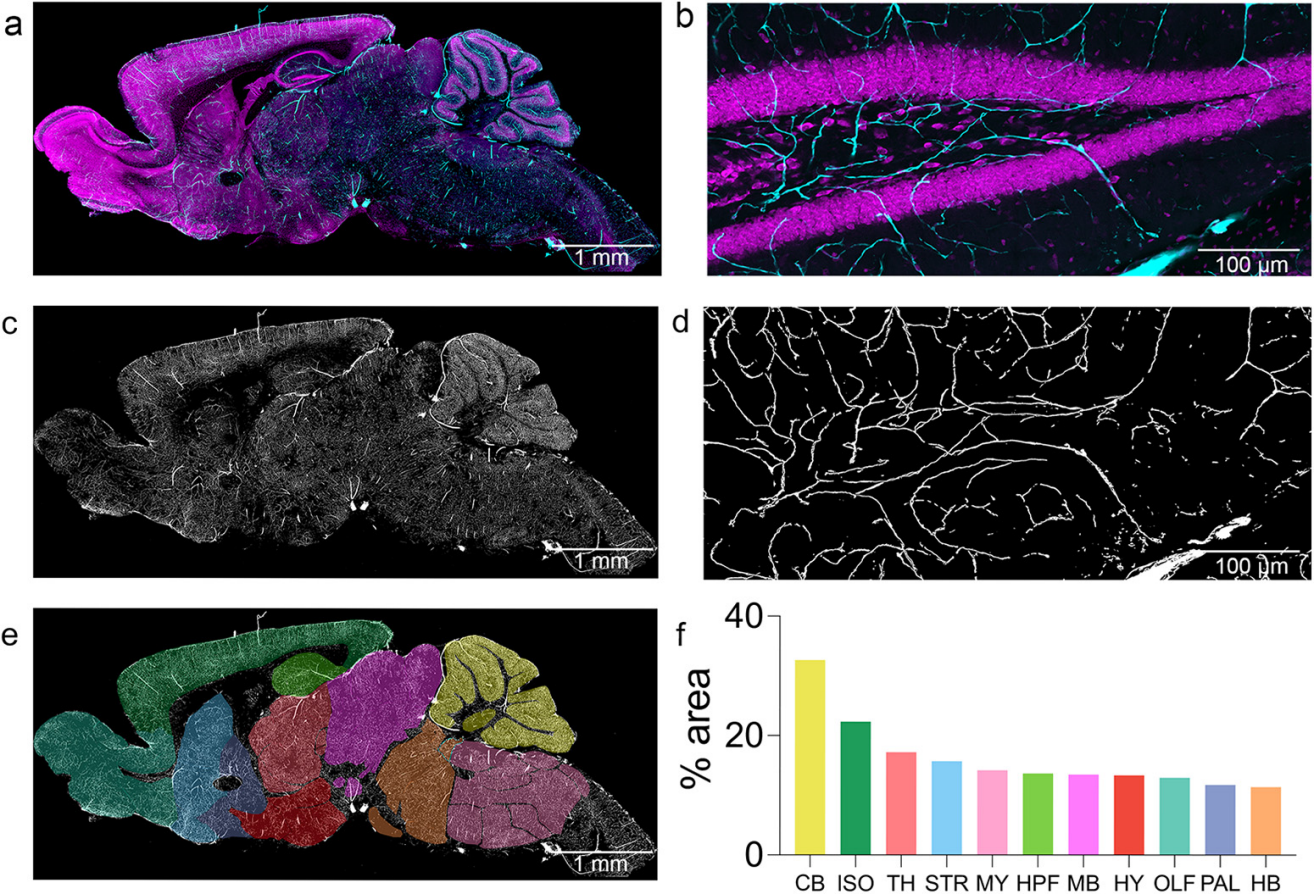
Assessing regional density of brain vasculature. Representative photomicrographs of vasculature in the adult mouse brain (***A***) and the dentate gyrus (***B***). FITC-perfused vasculature is labeled in cyan, while propidium iodide is displayed in magenta. Vasculature was segmented from background and binarized using *Ilastik* (***C***, ***D***). The image was registered to a custom atlas plate consisting of the isocortex (ISO), hippocampus (HPF), amygdala (AMY), olfactory bulbs (OLF), pallidum (PAL), cerebellum (CB), midbrain (MB), striatum (STR), thalamus (TH), hypothalamus (HY), medulla (MY), and hindbrain (HB; ***E***). Using a densitometry-based analysis approach, the regional density of vasculature was determined as a percentage of the overall region area (***F***). *Figure Contributions*: Dylan J. Terstege prepared the tissue, collected photomicrographs, and conducted the analysis.

Not all labels are suited for analyses based on object counts. It is often more useful to assess the distribution of vasculature in terms of the percentage of each region which it occupies. To facilitate this type of analysis, a “Volumetric Analysis” option was included in FASTMAP. Brain vasculature was binarized (Fig. 3*A–D*) and images were registered to a custom neuroanatomical atlas. The percentage of each region which was comprised of the vascular label was recorded (Fig. 3*E*,*F*).

### Amyloid distribution in CRND8 mice at different ages

Most label segmentation and atlas registration tools are used to determine the regional densities of a label. FASTMAP segmentation binarizes the labeled image using a pixel intensity threshold. Segmentation can be refined by restricting the range of accepted pixel intensities and the size range of accepted objects.

**Figure 4.**
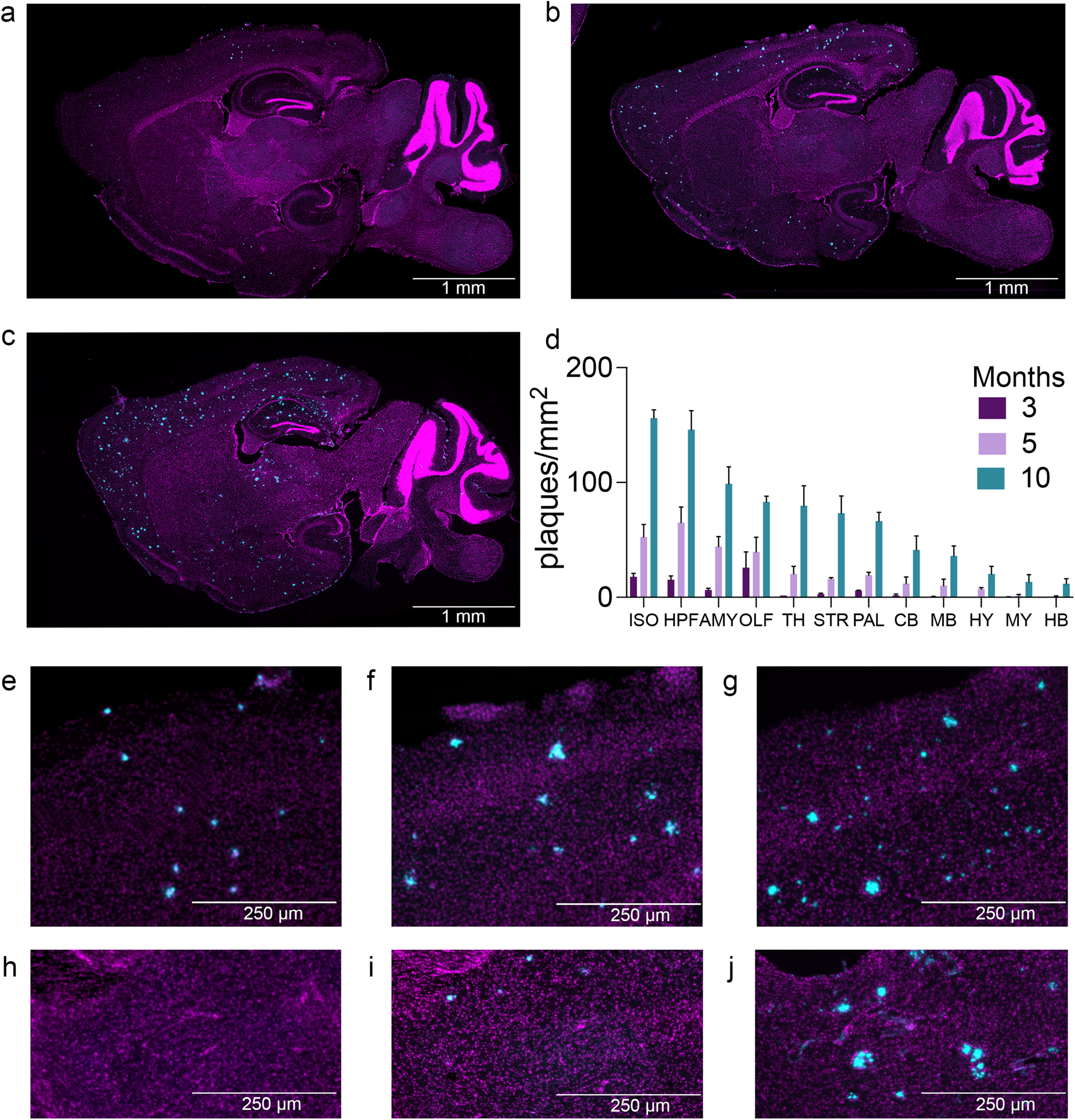
Mapping of age-related changes in amyloid plaque deposition density. Representative photomicrographs of amyloid pathology in 3-month-old (***A***), 5-month-old (***B***), and 10-month-old (***C***) CRND8 mice. Propidium iodide is displayed in magenta while the fluorescently labeled β-amyloid is labeled in cyan. ***D***, Amyloid density across the isocortex (ISO), hippocampus (HPF), amygdala (AMY), olfactory bulbs (OLF), pallidum (PAL), cerebellum (CB), midbrain (MB), striatum (STR), thalamus (TH), hypothalamus (HY), medulla (MY), and hindbrain (HB). High-resolution photomicrographs of the somatosensory cortex (***E–G***) and the reticular nucleus of the thalamus (***H–J***) further illustrate differences in amyloid pathology progression at 3 (***E***, ***H***), 5 (***F***, ***I***), and 10 (***G***, ***J***) months. Data presented as mean ± SEM. *Figure Contributions*: Dylan J. Terstege prepared the tissue, collected photomicrographs, and conducted the analysis.

To demonstrate the ability to efficiently calculate the regional density of a label of interest, we registered photomicrographs from groups of 3-, 5-, and 10-month-old CRND8 transgenic Alzheimer’s disease model mice to 12 major neuroanatomical regions and calculated amyloid density across the whole brain. We chose this experiment because the best demonstration of a tool such as FASTMAP is to show an ability to detect changes in marker expression in multiple regions across the entire brain. In these mice, the amyloid load is known to (1) become denser at a regional level over time, and (2) also becomes distributed throughout more regions across the brain with age. The contrast between native autofluorescence and the methoxy-X04-labeled amyloid was sufficient for the segmentation of the label of interest by ImageJ thresholding parameters. This comparison yielded insight into changes in plaque distribution and density (Fig. 4). Two-factor repeated measures ANOVA revealed significant main effects of age (*F*_(2,6)_ = 26.05, *p *=* *0.0011) and region (*F*_(2.880,17.28)_ = 66.45, *p *<* *0.0001). A significant interaction between age and region factors (*F*_(22,66)_ = 16.85, *p *<* *0.0001) indicated age and region dependent changes in amyloid plaque density in CRND8 mice. This is consistent with anticipated age-related changes in both amyloid deposition density and distribution in this transgenic line, supporting the application of FASTMAP to studies examining the regional densities of labels of interest.

### Atlas flexibility

Most tools for mapping tissue align images to standardized reference atlases of adult mouse, rat, or human brains. Standardized atlases are a useful tool for producing reproducible datasets. However, there are situations in which only a subset of regions is required, such as when analyses are being limited to a small collection of regions or when standard atlases have subdivided ROIs to a higher hierarchical level than necessary. In these cases, it becomes more efficient to register only the desired subset of regions, rather than the entire standardized atlas. We developed a tool which allows the user to curate their ROIs. This allows analyses to be efficiently tailored to specific experimental needs and facilitates the study of nontraditional tissue types, orientations, or regional organizations.

Custom atlas plate generation is a user-friendly process outlined step-by-step in the FASTMAP User’s Guide (Extended Data 1). Using reference images from existing atlases, users are instructed to trace ROIs in ImageJ before adding regions to the ROI Manager applet. Once all regions in the image have been traced, they are saved as an ROI set. This process is repeated until all desired atlas plates have been generated. Compiled custom atlases can then be shared during publication, and/or submitted to the FASTMAP GitHub repository for use by other researchers supporting transparency and replicability in image registration.

**Figure 5.**
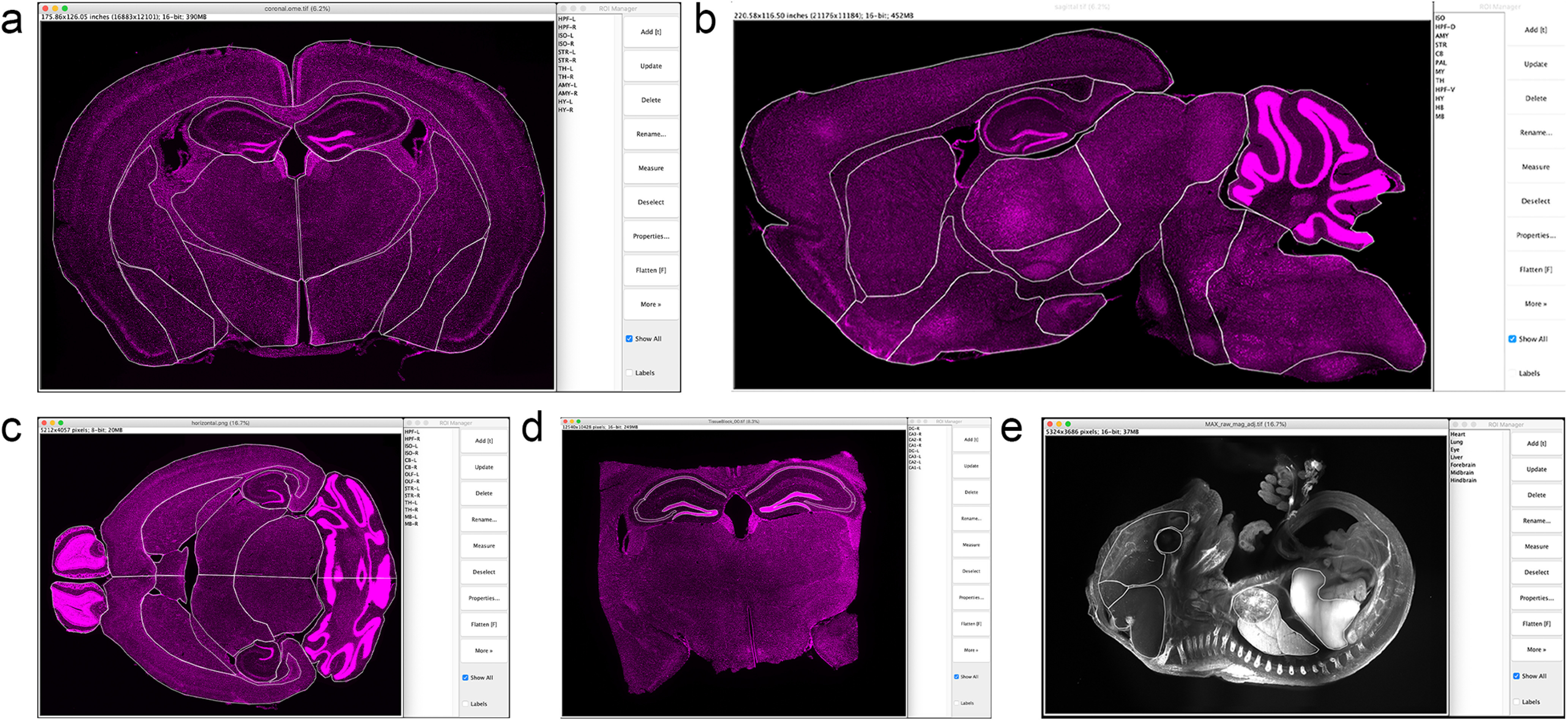
Compatibility of the registration tool with different tissue types, sample preparations, and imaging planes. Application of the registration tool to coronally sectioned adult mouse tissue (***A***), sagittally sectioned adult mouse tissue (***B***), horizontally sectioned adult mouse tissue (***C***), coronally sectioned tissue blocks of adult mouse tissue (***D***), and uDISCO-cleared e16 mouse embryo (***E***). *Figure Contributions*: Dylan J. Terstege prepared the tissue and collected the photomicrographs. Daniela O. Oboh prepared the custom atlas plates.

To demonstrate flexibility, we applied FASTMAP to a variety of tissue types and orientations (Fig. 5). Many other open-source tools only allow for tissue mapping in the canonical sagittal and coronal sectioning planes and most require a complete intact section as registration often involves an initial transformation based on the outline. In this demonstration, we covered these planes and extended on them by registering a mouse brain imaged in a horizontal plane. We also demonstrate here that partial sections, such as those that might be obtained as postmortem brain blocks from a brain bank can still be used with FASTMAP as the outline of the section is not required. Tools for the registration of mouse brains, and to a smaller extent rat brain, are becoming increasingly prevalent, these tools focus on adult brains. To demonstrate the register of other tissue types, the gross anatomy of an embryonic mouse was registered to custom atlas plates.

## Discussion

With the recent advances in imaging of brain-wide datasets comes the requirement for advances in the processing of these datasets. While considerable progress has been made in the development of tools for the mapping adult rat and mouse brains, many of these tools have specific applications with respect to tissue preparations, atlas organizations, and imaging plane orientations. To address the need for a simple yet flexible atlas registration tool, we have developed FASTMAP.

FASTMAP prioritizes flexibility and ease-of-use by allowing users to create atlas plates with custom region organization to suit the tissue type, preparation, and orientation used in their experiment. No advanced computing skills are required as the tool is fully integrated into a graphical user interface. The minimum technical specifications required to use FASTMAP are also considerably less than those of other registration tools, eliminating the need to purchase expensive workstations. Users are guided through the registration of their atlas plates to images which they have collected. Basic linear transforms scale the ROIs to the tissue section and then free-form manual transformations allow the user to correct regions individually to ensure registration accuracy in the face of nonuniform tissue expansion or shrinkage during processing or sectioning imperfections. User-friendly documentation, including atlas generation walk-throughs; image registration tutorials; low level (12 region) whole-brain mouse atlases in coronal, sagittal, and horizontal orientations; and a high level (63 region) whole-brain sagittal mouse atlas have been provided to help facilitate the application of this tool to datasets regardless of the user’s computational background (https://github.com/dterstege/FASTMAP/tree/main/Plates). Any additional user generated atlas plates developed in the future can also be hosted on the FASTMAP GitHub site.

To demonstrate the range of FASTMAP applications, we quantified c-Fos-positive cells and the densitometry of brain vasculature across custom neuroanatomical atlases. We then applied the tool to quantify age-related changes in amyloid density and distribution in CRND8 Alzheimer’s disease transgenic mice. Brain-wide quantification of amyloid plaque deposition across 12 neuroanatomical regions revealed both age and region dependent changes in plaque density. We found that the distribution of amyloid pathology increased with age, as regions with very little or no amyloid pathology at three months could show increased amyloid pathology in five-month-old mice. This increase was greater still in the 10-month-old group. These results align with existing understanding of the age-related development of amyloid pathology in this transgenic line, supporting the functionality of FASTMAP in the detection of labels of interest and the registration of images to a neuroanatomical atlas.

Segmentation and registration are instrumental to any atlas registration tool. In each example shown here, FASTMAP was able to align a reference atlas to the sample through linear transforms and minimal manual adjustments. Given the user-defined nature of the custom atlas plates, our tool can be applied to any image set. We have focused on registration of mouse brain images. FASTMAP, because of the ability to create custom atlas plates, can also be used with any species of interest. Other species commonly used in neuroscience such as rats, voles, hamsters, degus, marmosets, and zebrafish will work equally well with FASTMAP provided that a set of ROIs can initially be identified. This may be through the use of existing published atlas images or from *de novo* synthesis of atlas plates as needed. Additionally, our primary description of FASTMAP relates to brain registration but other biological systems may also be used.

FASTMAP has numerous potential uses but also some limitations. These limitations relate primarily to efficiency relative to some other tools in the processing of datasets with a high number of regions. Many atlas registration tools will fit hundreds of regions into their reference atlases. The manual adjustment of the same number of regions will take longer than the grouped transforms which are often applied in other tools. Many studies only require a handful of ROIs; these types of experiments will benefit most from the use of FASTMAP. While there is no maximum number of regions that can be registered with FASTMAP, there may be a maximum number that are feasible for a user to perform efficiently. This number will vary depending on the particular ROIs, their size and complexity as well as the users comfort level with and access to more complex analysis tools.

FASTMAP accuracy is ultimately dependent on the ability of the user to recognize neuroanatomical features for the ROIs. We reported high interrater reliability when assessing neuroanatomical region areas traced by independent raters who were familiar with rodent neuroanatomy. To achieve similar results, it is recommended that end users are well-versed in the anatomic features of their ROIs or refer to a reference atlas during atlas registration. Furthermore, while free-form manual correction allows the user to accommodate for imperfections during tissue processing, it is recommended that users use best practices when processing all tissues before image collection. Atlas familiarity and the quality of the histologic preparation are also limitations of most commercially available and open-source tools. Even automated registration tools must be checked for accuracy and an experimenter’s ability to do so is dependent on their aptitude to manually identify the ROIs. FASTMAP automatically saves fitted registration plates for later re-loading, providing the opportunity for quality control checks and for multiple cell type-specific markers to be used during repeated registration.

A limitation of many registration tools is the ability to segment labels based on morphology. Many tools refine segmentation based on combinations of pixel intensity, object size, and object sphericality. FASTMAP does not directly incorporate segmentation tools but instead interfaces with other tools in a flexible way that allows for enhanced and variable segmentation options. For example, the open source software *Ilastik* applies supervised machine learning to segment objects of interest based on properties of the pixels and objects in the images (Berg et al., 2019). This tool can be used to segment objects with complex morphologies and generate a binary output of these labels. These binary images can then be registered in FASTMAP. Other tools such as *CellProfiler* (McQuin et al., 2018) may also be used for this initial segmentation.

Given that manual adjustments are performed with FASTMAP there exists a possibility for experimenter bias to be introduced. However, scientific best practices of blinding experimenters to animal and group Identifications should prevent such problems. We have also incorporated into FASTMAP an option to export all generated ROIs projected on top of their registration channel. This allows for *post hoc* quality control checks and aligns with principles of open science as the entire registration data set can be uploaded to a data repository.

As the use of whole-tissue imaging to address diverse scientific questions becomes increasingly prevalent (Renier et al., 2014; Epp et al., 2015; Jing et al., 2018; Li et al., 2018; Pinheiro et al., 2021), it is imperative that analyses can flexibly meet the demands of these questions. Having noted the lack of a user-friendly and highly flexible registration tool, we developed an open-source tool to register images to custom atlases. We anticipate that the ease of use, versatility, and accessibility of our tool will facilitate many scientific questions.

## Synthesis

Reviewing Editor: Karen Davis, Krembil Research Institute Decisions are customarily a result of the Reviewing Editor and the peer reviewers coming together and discussing their recommendations until a consensus is reached. When revisions are invited, a fact-based synthesis statement explaining their decision and outlining what is needed to prepare a revision will be listed below. The following reviewer(s) agreed to reveal their identity: Laura DeNardo, Justin Kenney.

This is a nice, highly flexible tool that can be used for image segmentation. The fact that it integrates into ImageJ will make it easily accessible to many biologists. It’s lack of automation makes the throughput lower than other tools, but increases its ability to be adapted to a wide variety of image types.

1. The author’s have largely addressed my major concerns. However, I find the method by which they assessed the consistency and accuracy of the tool problematic. It looks like the way they are assessing consistency between individuals and between methods for segmentation (Figure 2) is by comparing the size of the regions identified. However, the same size/area of region can be obtained with very different shapes. It’s the extent to which different raters or approaches capture the same shape of a region that is important (for example, I can draw an elongated rectangle, a circle, or a square with the same area, but the shapes are very different). The reliability should be computed by comparing how much different segmentations overlap (e.g. percent overlap), not size/area.

2. The authors performed some experiments aimed to address reviewer concerns. It is nice to show in the new Figure 2 that the thalamic reticular nucleus can be reliable outlined with both DAPI, PV stain, and that FASTMAP performs similarly to NeuroInfo. What is being shown in panels c and d? Are they comparing individual human raters? This needs to be more clearly described in the figure legend. Also, it seems like they only analyzed one slice of a single brain in the experiments shown in Figures 1 and 2. Is this correct? If so, the authors should justify this. How did they choose the brain sections? Did they just pick the best one in terms of the cryosectioning quality? How much warping can FASTMAP tolerate? It’s difficult to tell how well it actually works based on what the authors have shown.

3. What do the different points in figure 2C represent? Are each of the data points from different animals? Why is there such large variability in the area of the RTN? The description in the results section is unclear as to what exactly was done here.

4. The presentation of Figure 2D makes it difficult to assess the author’s claims due to the large range of areas across the different regions (i.e. it’s difficult to see the 95% CI and where the Neuroinfo measure falls within that interval). However, if the author’s switch to using a more appropriate measure, like percent overlap (as suggested above), this may no longer be an issue.

5. It would be helpful/more convincing that the PV vs DAPI works as well, and the NeuroInfo vs FASTMAP by including an image that has an outline of the segmentations from different approaches, with the lines in different colors. That way the readers can see more directly how the shapes compare.

6. There are still some typos throughout. For instance, on line 194 “outlines” should be “outlined”.
