# Supplementary material for: FASTMAP: Open-Source Flexible Atlas Segmentation Tool for Multi-Area Processing of Biological Images
Source: eNeuro. 2022 Mar 16;9(2):ENEURO.0325-21.2022. doi: 10.1523/ENEURO.0325-21.2022 (PMC8938980; doi:10.1523/ENEURO.0325-21.2022)
Supplement: Extended Data 1 — FASTMAP user guide. Full FASTMAP user guide posted to the GitHub repository, at https://github.com/dterstege/FASTMAP. Download Extended Data 1. Download Extended Data 1, ZIP file. [file enu-eN-OTM-0325-21-s02.zip › Extended Data 1/FASTMAP_USER_GUIDE.pdf]

FASTMAP

Flexible **A**tlas **S**egmentation **T**ool for **M**ulti-**A**rea **P**rocessing

User's Guide
V 1.1.1
October 2021

TABLE OF CONTENTS

INTRODUCTION -- 3

INSTALLATION INSTRUCTIONS ----------------------- 4

IMAGE PROCESSING TUTORIAL ---------------------- 5

ATLAS PLATE CUSTOMIZATION ---------------------- 14

ROI OVERLAY -- 17

TROUBLESHOOTING --------------------------------------18

CITATION ---18

CONTACT US -- 18

INTRODUCTION

FASTMAP (**F**lexible **A**tlas **S**egmentation **T**ool for **M**ulti-**A**rea **P**rocessing) is a tool for the registration of biological images to custom atlas plates and the segmentation of labels of interest within atlas regions. This tool operates as an ImageJ Plugin that draws upon versatile and powerful image analyses tools in ImageJ and presents them as a clean, concise, and easy to follow plugin.

Through linear scaling and free-form adjustments, FASTMAP users can flexibly register biological images of any tissue type, orientation, or preparation for which they have drawn custom atlas plates.

FASTMAP was created by [REDACTED], a Neuroscience PhD candidate in the [REDACTED] at the [REDACTED]

INSTALLATION INSTRUCTIONS

FASTMAP was designed with ease-of-use at the forefront of our minds. The installation process reflects this, with a simple 3-step approach:

1. **Install ImageJ**. Ensure that ImageJ is installed. If not previously installed, it can be downloaded here.

2. **Download the Appropriate Version of FASTMAP.** Ensure that the version of FASTMAP which you are downloading is appropriate for your operating system.

   FASTMAP is currently available for:

   [REDACTED]

   [REDACTED]

3. **Install Plugin in ImageJ.** Before installing, see the settings subsection of the Image Processing Tutorial to determine whether the plugin should be modified in any way to best suit your project prior to installation.

   Once satisfied with the settings, open ImageJ and select "Plugins > Install...". Navigate to the newly downloaded version of FASTMAP and allow this to save to the ImageJ Plugins folder.

   Note: If installing on FIJI, ensure that you use the "Install..." command rather than the "Install Plugin" command.

IMAGE PROCESSING TUTORIAL

The following guide will outline how to process a dataset using FASTMAP:

INITIALIZATION:

1. **Images.** FASTMAP has been optimized for 8- and 16-bit .tif files, with the label of interest being in an image file which is separate folder from the channel to be used as a reference during atlas registration (DAPI, Propidium Iodide, Autofluorescence, etc.).

2. **File Organization.** Files should be organized under a common "parent folder". This parent folder should contain at least two subfolders: one containing the "label images" and a second containing the "registration images". Folders can contain all images for an entire project, so long as the images for each subject are in continuous strains and corresponding files appear in the same alphabetical order in both folders.

3. **Atlas Organization.** FASTMAP is highlighted by its applicability to a nearly limitless range of biological samples, irrespective of sample type or orientation. This is due to the flexibility of the atlas plate registration and generation. To facilitate immediate use, [REDACTED].

IMAGE PROCESSING TUTORIAL

RUNNING THE PLUGIN:

1. **Running the Plugin.** With image files and atlas plates in the proper formats and organizations, the plugin may now be ran. Open ImageJ, select “Plugins > FASTMAP”. The user will be to navigate to the parent folder containing both image channels. A second prompt will ask the user to navigate to a folder containing the desired atlas plates for the analysis.

2. **Image Assignment.** A dialogue window titled FASTMAP will populate and prompt the user to identify which of the subfolders in the parent folder contains the registration images and which contains label images.

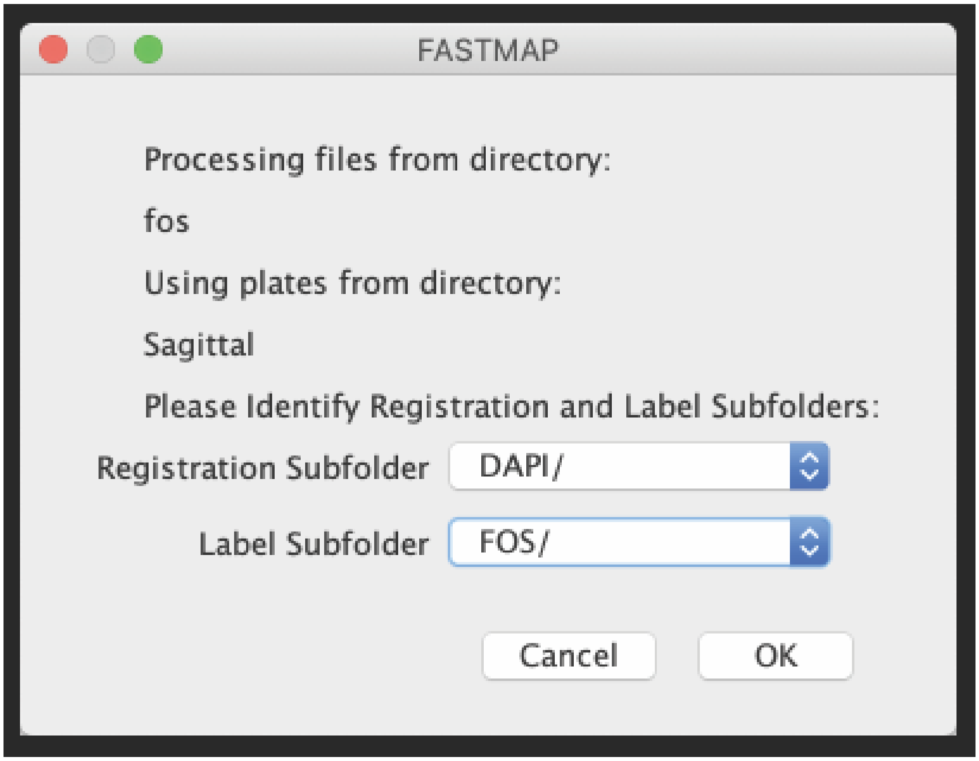

3. Range and Analysis Type. The plugin will then display the number of files in the registration and label subfolders. Output data will be appended based upon the range inputted during this step, so if images from multiple samples are in the same folder the range of the current sample should be defined.

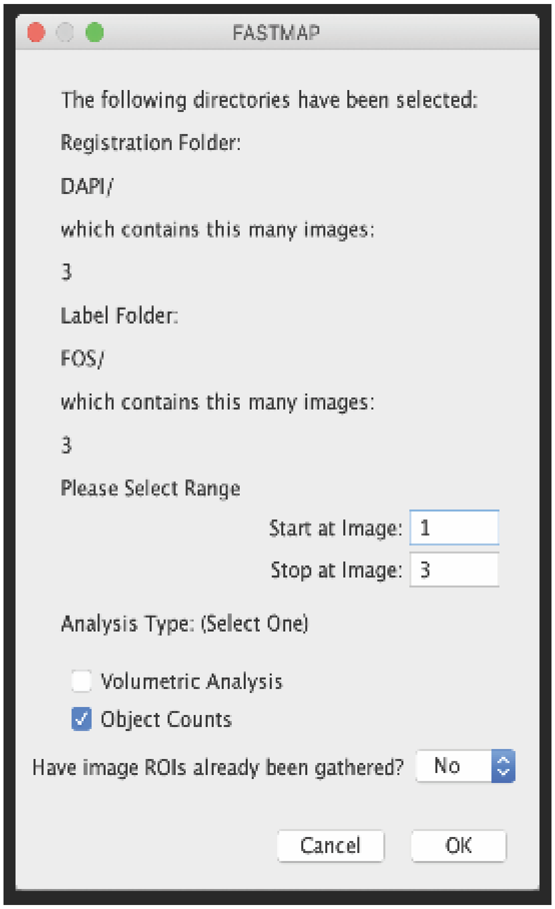

This window also asks which type(s) of analysis should be applied to the image set. Analysis types are as follows:

Volumetric Analysis: reports the total area of the region, the summed area of all labels within the region, and the percentage of the region that is comprised of labels.

Object Counts: reports the total area of the region and the number of objects counted within each region.

Both the volumetric analysis and the object counts analysis types have been written to use the default auto-thresholding parameters of ImageJ. If working with already binarized labels as inputs or raw images in which the default thresholding is insufficient, see the settings section for information on how to modify the plugin to better suit the input images.

At the bottom of this window, the user will be asked whether ROIs have already been gathered for this range of images. If images have previously been processed through FASTMAP and the ROIs have been saved, this option can be chosen to conserve the previously registered ROIs. For more information on saving ROIs, click here.

4. Choose Registration Plate.

A composite image displaying all plates from the selected atlas will populate on the screen. Drag this to the right side of the screen before selection 'OK', otherwise it will be hidden by the next image which will populate on the screen. This image will be the working registration image with a prompt asking which plate the image most closely resembles.

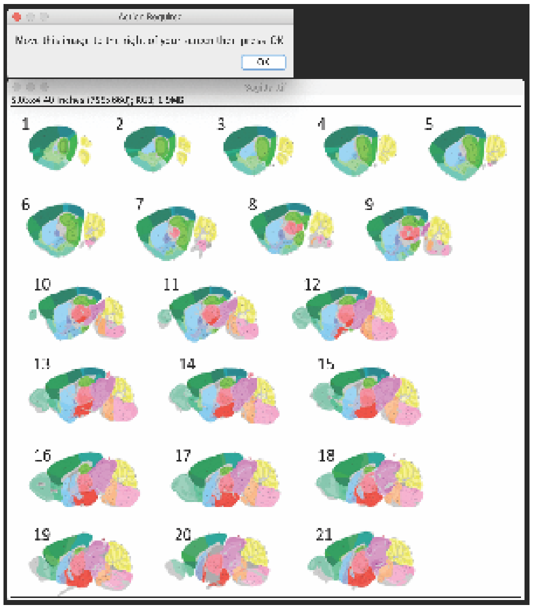

5. **Automated Resizing.** FASTMAP can be applied to images collected using a wide variety of microscopy techniques. Furthermore, it can be applied to images of different sizes and resolution. If the user is looking at the same group of regions using multiple microscopy types of magnifications, the plugin can scale atlas plates to limit the extent to which regions need to be manually resized and manipulated. This linear transform is initiated by drawing a rectangle around the sample in the image.

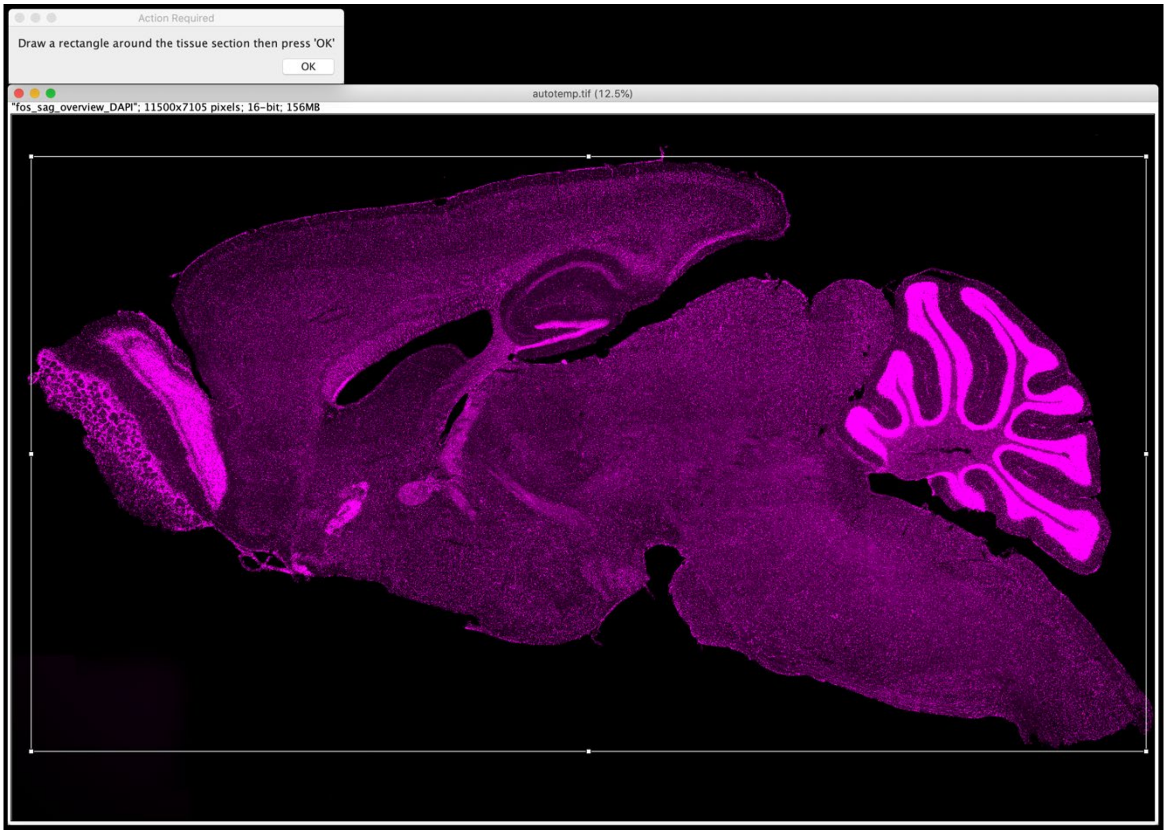

6. **Adjusting Registration.** One-by-one, regions will resize and come to the center of the image. Move, adjust, or delete the ROIs as needed – clicking 'OK' in the dialogue window once satisfied with each region. There is no need to adjust any "dummy" regions (see GitHub for examples), but do not delete these regions until after all regions have been adjusted.

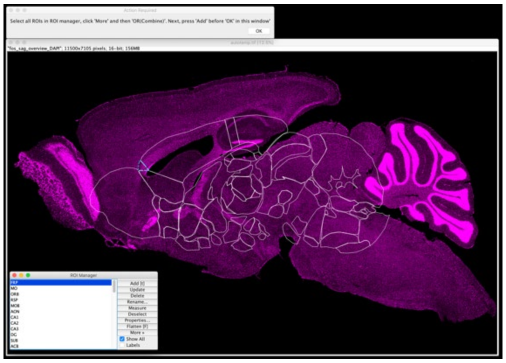

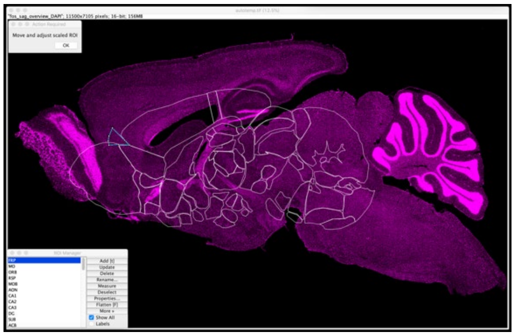

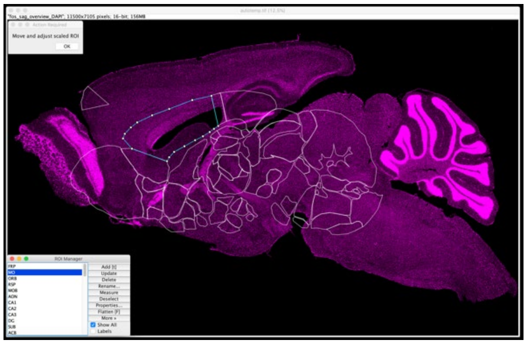

With all regions adjusted, any ROIs which are not essential to the analysis may now be deleted from the ROI Manager.

SAVING:
After adjusting all ROIs, FASTMAP will automatically save the ROI set under the name of the registration channel image in a folder titled 'RegisteredPlates' in the atlas plate directory. This will allow for the adjusted ROIs to be called upon at a later date should the user wish to apply different thresholding parameters or a different analysis type to the image.

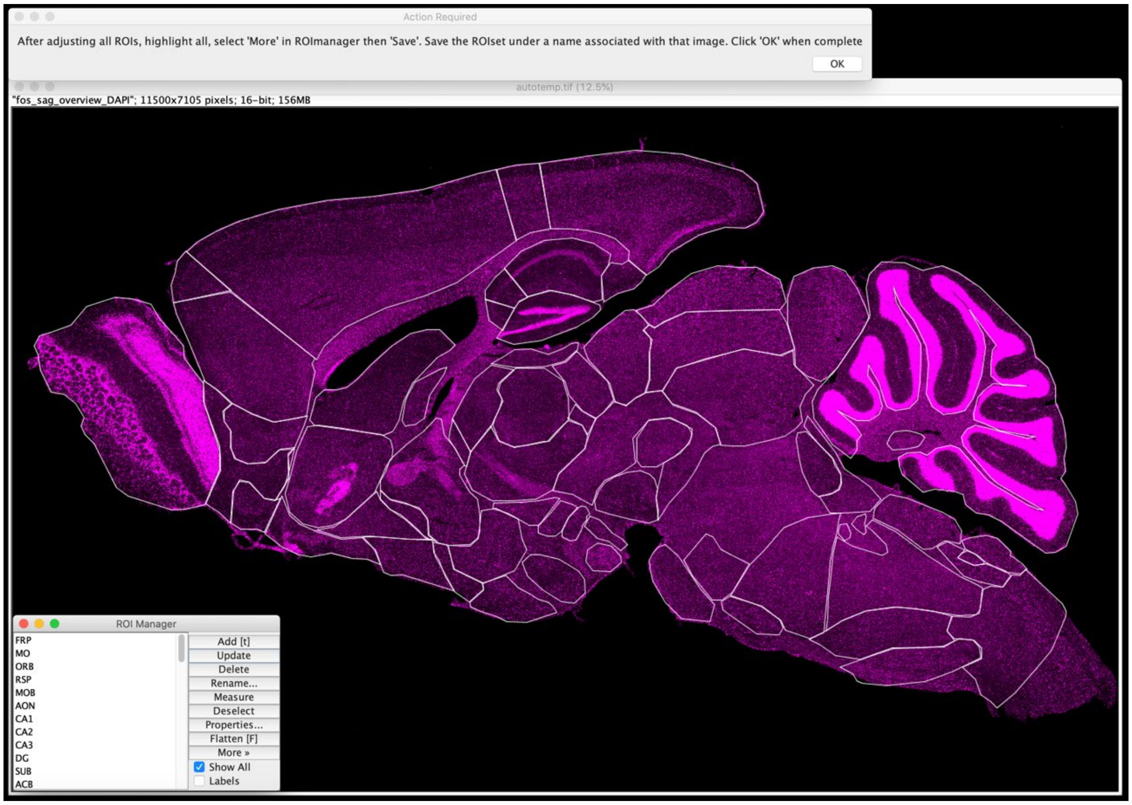

7. **Settings.** There are a few settings which may be customized to best suit particular sets of images. These can be adjusted by dragging and dropping the .ijm file into ImageJ and editing lines as needed. The default settings upon download are set to:

    Thresholding: "Default"
    Threshold Background Colour: "Dark"

    These settings are found in both the volumetric analysis and the object counts sections of the code and should be adjusted according to the analysis type.

    Note: if using an already binarized image, the following lines can simple be commented out using "//":

```
setAutoThreshold("Default dark"); //may comment out this line if using an already binary image
run("Convert to Mask"); //may comment out this line if using an already binary image
```

8. **Outputs.** After all images from the specified file range have been processed, output files will appear in the parent folder. Depending on the analysis type, they will either be named *VolumetricAnalysis.csv* or *RegionalCounts.csv*.

   Note: There is no sample identifier in this file name, so it is advised that the filename is edited before the user starts processing the next range of images to avoid overwriting the previous file

ATLAS PLATE CUSTOMIZATION

A benefit of FASTMAP over other image registration tools is its atlas flexibility. With minimal up-front tracing, atlases can be generated for application to any image set.

Sample plates can be found [REDACTED].

The process of generating a custom atlas is outlined in the following steps:

1. **Collecting Reference Images.** All atlas images should be based on replicable references. These could be a subset of regions in an existing atlas or clearly delineated regions specific to the image set of interest. These images do not need to be of any particular format or resolution but should be clear enough for easy tracing of regions of interest.

2. **Region Tracing.** Open the first reference image using ImageJ. Using the Polygon Tool, trace a region of interest. By hitting "t" on the keyboard, add this ROI to the ROI Manager applet. Rename this newly logged ROI with the name of the region.

Tips for region tracing:

- *Clicking "Show All" in the ROI Manager applet is recommended.*
- *Ensure that regions do not overlap during the initial tracing*
- *If ROIs do not cover the entire X and Y range of the sample, add a dummy ROI as a space filler. This will ensure that regions resize correctly when running the plugin. For an example of this, see the* [REDACTED].

Continue tracing and renaming until all regions of interest present in the reference image have been traced.

3. **Saving Plates.** With all regions traced, highlight all ROIs in the ROI Manager applet and click "More > Save". Plates should be saved to a folder designated to that particular atlas as "RoiSet_xx.zip", with xx being an identifier unique to that particular plate. Leading zeros are not required for single digit identifiers.

   With all regions saved, the current reference image can be closed, and the next reference image can be loaded. ROIs aligned to the previous reference image can then be modified, deleted or added to the list as needed.

ATLAS PLATE CUSTOMIZATION

4. **Composite Atlas Image.** When running the plugin, a composite image of all the plates in the selected atlas populates on the screen and the user is asked to identify which plate most closely aligns with the image they are registering. This composite image can be generated in a variety of programs (*Adobe Illustrator*, *Microsoft Office's PowerPoint,* and *InkScape* all work well for drafting composite images). Some important notes with this composite are the following:

   - Composite images must be in .tif format. Images can initially be generated as a .PNG or .JPG before being converted to .tif in ImageJ.

   - Plate identifiers listed on the composite image should align to the identifiers assigned to the RoiSet zip files during atlas generation.

   - The name of the composite image file must match the name of the atlas folder. For example, the sample sagittal atlas is in a folder called "Sagittal_HighLevel", therefore the composite image should be named "Sagittal_HighLevel.tif".

ROI OVERLAY

When demonstrating atlas registration, it is often useful to provide images showing exactly what was registered. In saving all ROI sets after registration, FASTMAP facilitates this process. All that users need to do is load the registered ROI set and flatten the overlay onto the appropriate target image

To streamline this process, I've created BRO: Batch ROI Overlay. BRO asks users to identify a target image folder and a folder containing their registered ROI sets and then outputs a PNG with the merged image.

BRO can be found on GitHub [REDACTED]

TROUBLESHOOTING

Troubleshooting section will be updated periodically should common issues start to arise with the plugin. See [REDACTED] for the most up-to-date information.

CITATION

If you find FASTMAP to be useful, and wish to apply it to your research, we ask that you cite the following article outlining this flexible open-source atlas registration tool:

CONTACT US

If you have any further questions or concerns about FASTMAP, the author(s) can be reached as follows:

[REDACTED]
Development / Conceptualization / Documentation / Validation / Corresponding Author
[REDACTED]
[REDACTED]

[REDACTED]:
Atlas Generation

[REDACTED]:
Conceptualization / Principal Investigator
[REDACTED]
